# Filling a void? The role of social enterprise in addressing social isolation and loneliness in rural communities

**DOI:** 10.1016/j.jrurstud.2019.01.024

**Published:** 2019-08

**Authors:** Danielle Kelly, Artur Steiner, Micaela Mazzei, Rachel Baker

**Affiliations:** Yunus Centre for Social Business and Health, Glasgow Caledonian University, M201 George Moore Building, Cowcaddens Road, Glasgow, G4 0BA, United Kingdom

**Keywords:** Social isolation, Loneliness, Social enterprise, Health, Wellbeing

## Abstract

Social isolation and loneliness has been classed as a major public health concern due to its negative physical and mental health implications, and living in a remote or rural area is a prominent contributing risk factor. Community-led social enterprise models are recognised in government policy as a potential preventative measure for social isolation and loneliness, yet there is a lack of understanding of their application in rural contexts. The objectives of this paper are to investigate the role of social enterprise in addressing social isolation and loneliness in rural communities, and to explore the pathways in which social enterprise activity may act upon the health and wellbeing of social enterprise beneficiaries. We also discuss the capacity of rural community members to deliver and sustain such services. The study used in-depth interviews over a three-year period with 35 stakeholders from seven social enterprises in the Highlands and Islands of Scotland, including board members, staff, volunteers and service users. Findings showed that social enterprises are successfully providing activities that counteract factors contributing to social isolation and feelings of loneliness, leading to wider health and wellbeing benefits for individuals. However, the sustainability and continuity of social enterprises are questionable due to the burden on smaller populations, limited expertise and knowledge of running social enterprises, and effects on the personal lives of social enterprise volunteers and staff. This study supports suggestions that social enterprises can be generators of health and wellbeing through their varied remit of activities that impact on the social determinants of health. However, it also shows that relying on social enterprise as a particular solution to social isolation and loneliness is precarious due to complexities associated with rurality. Therefore, rural policy and practice must move away from a ‘one size fits all’ approach to tackling social isolation and loneliness, recognise the need for local level tailored interventions and, through harnessing the potential or rural social enterprises, enable flexible service provision that correlates with rural context.

## Highlights

• Rural social enterprise activity is impacting on factors that contribute to social isolation and loneliness.
• Rural social enterprises provide services that generate opportunities to gain social support and form social relationships.
• As a result, rural social enterprises have a positive impact on the health and wellbeing of social enterprise stakeholders.
• Social enterprise service provision also has negative impacts on the health and wellbeing of staff and volunteers.

## 1 Introduction

Social isolation and loneliness have been classed as major public health concerns due their negative health and wellbeing implications (Klinenberg, 2016; Public Health England, 2014; Nyqvist et al., 2016). Social isolation is described as the ‘quality and quantity of the social relationships a person has at individual, group, community and societal levels’ (Scottish Government, 2018a:5), whereas loneliness is an individual's subjective feeling about the level of social relationships that they may have (De Jong-Gierveld et al., 2006). Further distinctions are made between situational loneliness, a phase of loneliness which may occur after a particular life event, such as bereavement; and chronic loneliness, a long-term state in which the individual has a continued inability to form satisfactory social relationships (Hart, 2016; Shiovitz-Ezra and Ayalon, 2010). The absence of social relationships and support networks has been found to impact on individual's social wellbeing, leading to decreased levels of happiness, increased anxiety and lowered self-esteem (Bernard and Perry, 2013; Cacioppo et al., 2006; Coyle and Dugan, 2012; Greaves and Farbus, 2006). Further, physical effects of social isolation and loneliness can be seen through increased susceptibility to coronary heart disease and stroke (Valtorta et al., 2016), mortality (Holt-Lunstad et al., 2010; Laugesen et al., 2018), and dementia (Holwerda et al., 2014). Older people are commonly recognised in literature as a prominent risk group for social isolation and loneliness, due to factors related to ageing, such as the loss of friends and family, decreased mobility, and poor physical health (Bernard and Perry, 2013; Cattan et al., 2005; Gardiner et al., 2018). Young people, on the other hand, have been found to be socially isolated through unemployment and low education levels (Matthews et al., 2016, 2018).

Common factors contributing to social isolation and loneliness are poor transport links, poor health, being an informal carer or being a single parent (Hart, 2016; Public Health England, 2015; McCann et al., 2017). Living in a remote or rural location has been found to be a substantial risk factor for social isolation and loneliness (Henning-Smith et al., 2018; Hart, 2016), with many rural residents experiencing intense and chronic feelings of loneliness (NHS Highland, 2016). Further, large proportions of older people commonly reside in rural locations, who have been shown to be more likely than the rest of the population to experience social isolation. Risk factors can be exacerbated by the inherent geographical challenges in accessing services due to inflexible or limited public transport links (Hart, 2016; NHS Highland, 2016). Rural communities are also typically more sparsely populated than their urban counterparts, and there can be a lack of local amenities and facilities that facilitate social interaction (Farmer et al., 2011). While literature has described the causes of social isolation and loneliness in rural contexts, there is a scarcity of studies that have explored the ways in which social isolation and loneliness can be prevented and reduced in remote and rural settings.

Taking this into consideration, the objectives of this study two-fold:

(1) To investigate the role of social enterprise in addressing social isolation and loneliness in rural communities;
(2) To explore the pathways in which social enterprise activity may act upon the health and wellbeing of social enterprise beneficiaries.

In our findings we also further explore the capacity of rural community members to deliver and sustain social enterprise services.

## 2 Background

### 2.1 Social isolation and loneliness policy context

Research and policy reports officially recognise the negative impacts of social isolation and loneliness on health and wellbeing internationally (Finlay and Kobayashi, 2018; Franck et al., 2016; Marmot, 2010). Government supported campaigns to reduce social isolation and loneliness have been launched in the UK, Denmark and Australia (Jopling, 2015; The Mary Foundation, 2018; Wyatt and Marino, 2018). The Australian Government has committed 46.1 million dollars to support local volunteer organisations to set up visitor and befriending schemes to tackle loneliness in older adults (Wyatt and Marino, 2018), whilst in Denmark, programmes have been set up piloting educational materials and activities to improve social networking amongst adults, both young and old (The Mary Foundation, 2018).

In the UK, social isolation and loneliness have been recognised as a major priority for the delivery of adult social care (HM Government, 2007), and the UK Governments and national public health bodies are committed to creating strategic measures for its alleviation (HM Government, 2012; Public Health England, 2014, 2015). Moreover, there is a commitment to identify types of interventions that could reduce social isolation and loneliness, and improve health and wellbeing (Public Health England, 2015). More recently, the Scottish Parliament launched an enquiry through the Equal Opportunities Committee, resulting in the creation of, amongst many, a national strategy, marketing and publicity campaigns, and a call for further research into prevention measures for social isolation and loneliness (Scottish Parliament, 2015). The enquiry also called for evidence of how the third sector may be addressing social isolation and loneliness. In particular, recognition was given to the potential role of community-based social enterprise^1^ models in providing support in areas not met by the public sector (Scottish Government, 2018a), such as the provision of community transport and activities that encourage social connections (Senscot, 2017). Nonetheless, there is little evidence to suggest social enterprise has the capacity of providing alternative services for healthier communities (Roy et al., 2014; Macaulay et al., 2018), particularly in rural community settings.

Although social isolation and loneliness has gained widespread government attention, what has been presented so far is a ‘one-size fits all’ approach to prevention across both urban and rural contexts. There has been an emphasis on ‘empowering communities to tackle social isolation and loneliness’, yet little consideration of the capacity of rural communities to provide localised solutions (Scottish Government, 2018a:13). Living in a rural area may have been recognised by the state as a risk factor for social isolation and loneliness, amongst many, in public health and policy reports (Scottish Government, 2018a; McCann et al., 2017), however, there has been little consideration of the complexities of rurality and what exactly influences individual experiences of social isolation and loneliness in rural and remote environments (Lilburn, 2016). Moreover, there is a lack of evidence of the existing work that social enterprises are doing in rural environments that might support its promotion as a definitive solution.

### 2.2 Social enterprise and health

Although evidence is lacking on the ability of social enterprise to deliver primary health and social care (Caló et al., 2018; Heins et al., 2010), it has been suggested that social enterprise can be a generator of general health and wellbeing benefits to communities through their varied remit of activities (Nyssens, 2007, 2014; Ridley-Duff and Bull, 2015; Roy et al., 2014; Wyper et al., 2016). There is a well-established literature showing that health and wellbeing can be impacted by social determinants of health, such as unemployment and poor access to transport (Bambra et al., 2010: Dahlgren and Whitehead, 2006). Therefore, social enterprise activities and service provision, such as social care, employment and community transport, could positively impact on these upstream social determinants of health (Roy et al., 2014).

At this stage, links between social enterprises and health are primarily conceptual with limited empirical evidence of their health and wellbeing outcomes. It has been hypothesised that different social enterprise processes may have multiple impacts on different actors, such as staff and service users, both directly and indirectly, whether they are explicitly health related services or not (Macaulay et al., 2018; Roy et al., 2014). Such conceptualisations provide a ‘platform’ from which future supportive evidence on the health benefits of social enterprise activity is required (Macaulay et al., 2018: 757). Early findings have pointed to the psychological and physiological impacts of social enterprise activity on individuals, such as increased self-esteem and confidence, social capital, improved nutrition, and improved health seeking behaviours (Caló et al., 2018; Macaulay et al., 2018; Muñoz et al., 2015). Nonetheless, the impact of social enterprise activity on social isolation and loneliness, particularly in rural settings, has yet to be explored.

Social enterprises are often seen as more pro-active than the state at meeting social needs as they are commonly rooted within communities and can offer more flexible alternative or complementary interventions to statutory services (Nyssens, 2007; Roy et al., 2013; Scottish Government, 2018a). In this way, social enterprises may be well suited to rural contexts where innovative and specialist community solutions are often required to counteract the withdrawal of and limited access to services and facilities (Steiner and Teasdale, 2018). However, questions can arise around the capacity of communities to sustain social enterprise activities in rural contexts.

### 2.3 Rurality and social enterprise

Rural populations face challenges of geographical isolation, communication, transport issues and retention of populations (Farmer et al., 2008; O'Shaughnessy et al., 2011; Steinerowski et al., 2008). Rural communities are also more vulnerable than their urban counterparts to public finance cuts and the withdrawal of public services, such as transport and local health services, due to the high cost per head of population to deliver services in sparsely populated areas (Hodge et al., 2017). In terms of healthcare, this means that individuals need to travel large distances to towns and cities to access physical and mental health services (Dixon and Welch, 2000). To counteract this, communities are increasingly encouraged to co-produce their own local level services, such as community transport, shops and community centres (Markantoni et al., 2018; Steiner and Teasdale, 2018).

Rural communities are typically described as harvesting a collective resilience and drive to sustain in the face of economic, social and environmental hardship (Skerratt et al., 2012; Steiner and Markantoni, 2013). In particular, high levels of volunteerism and civic participation in rural areas can be viewed as counteractive to service withdrawals (Scottish Government, 2015; Skerratt et al., 2012; Osbourne et al., 2004). For this reason, rural communities can be viewed as the perfect breeding grounds for social enterprise activity that solves existing problems and contributes to their long-term sustainability (Kay, 2003; Shucksmith et al., 1996). Indeed, rural citizens are more likely than their urban counterparts to be involved in social rather than commercial entrepreneurship (Social Value Lab, 2017). Nonetheless, rural communities often have limited pools of human and economic resources to draw from. For that reason, there can be a shortage of entrepreneurial knowledge and skills to develop social enterprise activity, and an overburden on community volunteers (Munoz et al., 2015; Munoz and Steinerowski, 2012). Furthermore, there can be limited access to business markets and ability for economic growth of social enterprises due to geographical factors, low paid labour markets and low-level incomes (Munoz et al., 2014; Steinerowski et al., 2008; Steinerowski and Steinerowsksa-Streb, 2012). For these reasons, the sustainability of rural social enterprise requires an understanding of local needs and context to distinguish the level of support and nurturing that is required (Skerratt, 2013).

Considering the presented aspects of the social isolation and loneliness policy context and the role of social enterprise in rural health and care service provision, this paper reports on findings related to the impact of social enterprise activity in addressing social isolation and loneliness in rural communities. The findings presented are from ‘Growth at the Edge’, a primary empirical study of rural, community-based social enterprises in the Highlands and Islands of Scotland. This project was a component of a wider ‘CommonHealth’^2^ research programme, funded by the Medical Research Council (MRC) and Economic and Social Research Council (ESRC). This wider programme aimed to develop methods to evaluate new pathways to health creation and health inequalities reduction arising from social enterprise activity. More broadly, the programme has been a novel attempt to conceptualise the activities of social enterprises and their impacts on the social determinants of health. This has involved seven individual projects exploring differing social enterprise sectors, including housing, work integration and the arts; and across a wide geographical area of Scotland, including rural communities.

## 3 Methodology and methods

### 3.1 Study context

**Fig. 1:**
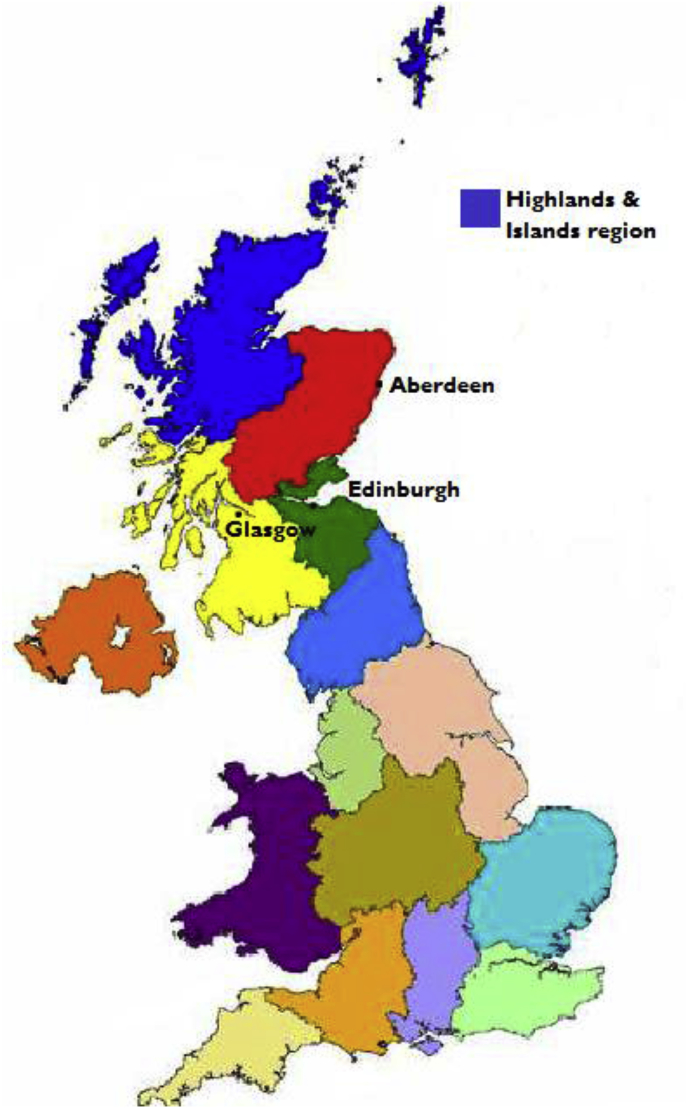
Map showing the Highlands and Islands region of Scotland, UK.

Scotland, in the UK, has a population of just fewer than 5.3 million people, 20% of which live in remote and rural areas (MacVicar and Nicoll, 2013; Scottish Government, 2015). Our research focused on the Highlands and Islands region of Scotland (Fig. 1), one of the most sparsely populated of Europe, with a population size of approximately 466,000 (Highlands and Islands Enterprise, 2014). This area is characterised by high levels of out-migrating youth who leave small communities for employment and education opportunities in urban cities (Stockdale, 2006, 2016), and a high proportion of residents over 65 years old - a group of people who are more likely to experience social isolation and loneliness (Farmer et al., 2010, 2011; Levin and Leyland, 2006). Recent survey conducted by the NHS Highland (2016), showed that two thirds of the study participants aged 65 years and over reported being lonely, in particular, those living alone in a remote area or those living with a long-term health problem or disability. High levels of depression have also been found in populations living in remote areas of Scotland, with evidence showing geographical isolation and distance to mental health facilities as contributing factors (Skerrat et al., 2017). Recent figures have shown that 34% of all social enterprises in Scotland are located in rural areas, with 21% of social enterprises existing in the Highlands and Islands of Scotland (Social Value Lab, 2017). This equates to an average of four social enterprises operating per 1000 people in rural areas, compared to one per 1000 people in urban areas (Social Value Lab, 2015), making rural Scotland a relevant case study area when investigating the role of social enterprise in addressing social isolation and loneliness in rural communities.

### 3.2 Social enterprise sampling and sample characteristics

A list of 168 registered social enterprises in the study location was provided by Highlands and Islands Enterprise, the Scottish Government economic and community development agency for the region. From this list, 21 social enterprises were shortlisted based on the inclusion criteria of (i) being based in a remote/rural location as defined in the Scottish Government urban/rural classification (Scottish Government, 2018b); (ii) having a significant sample size of no less than 10 engaged community members; (iii) having frequent, ongoing activity (not intermittent) within the community, and (iv) not directly related to health or providing a specific health service in their remit. From this, a maximum variation sampling technique was used to cover a range of social enterprise sector types and geographical locations across the Highlands and Islands. Social enterprises were then scoped to determine their availability and to take part in the project over a 3-year period. From this inclusion exercise, seven social enterprises were chosen to take part. The details of each social enterprise are shown in Table 1.

**Table 1:** Social enterprise included in the study, activities provide/used and geographical area served.

Social enterprise	Social Enterprise Activities	Participants of social enterprise	Area/Population served	Alternative services
**Seaboard Hall**	Community hub/café, leisure centre, entertainment, tourism centre	Staff: Running of café, organisation of events, administration Volunteers: Board membership, running community activity groups Service users: attending café, events, training, social/leisure activities	Providing centralised services for people of all ages from 3 villages with a combined population of approximately 1027 people (Highland Council figures for 2016/2017).	Nearest alternative service 8 miles away in closest town with limited public transport links.
**Cothrom, South Uist**	Education and training centre with on-site nursery and recycle centre	Staff: Providing training and education, administration Volunteers: Board membership, putting on events, running workshop/upcycling Service users: Receiving education and training, taking part in social/leisure activities	Providing services for all ages. Serving a geographical are including 4 remote islands in Outer Hebrides with a combined population of approximately 4167 people (Scottish Census Fig. 2011).	Only alternative education and employment training centre is on the mainland (4 h away by ferry boat).
**COPE Ltd, Shetland**	Employment and skill development for adults with learning disabilities (garden centre, recycling centre, catering business, soap production)	Staff: Providing administration, human resources and management Volunteers: Board membership, working in departments to assist service users and staff Service users: Taking part in employment, training and events, and social/leisure activities	Service provision inclusive of adults with learning disabilities (aged 18 + years) across the entire Shetland Islands (1466 km^2^). Approximately 147 recorded people have adult learning disabilities on the Shetland Isles (Scottish Commission for Learning Disabilities, 2017).	Only alternative employment and skills development for adults with learning disabilities on the mainland (1-h flight or 15 h ferry).
**Transport for Tongue (T4T), Tongue**	Community transport initiative (transport to services and amenities in urban areas)	Staff: Providing operations support, administration, drivers paid hourly Volunteers: Board membership, some volunteer drivers Service users: Using community bus and car services to reach amenities	Providing transport for residents of all ages across 3 villages to travel up to 90 miles away. Population of Tongue 564 people, population of Melness and Skerray less than 150 people (Scotland's Census, 2011).	No alternative transport service due to withdrawal of public transport links for that area.
**Atlantic Islands Centre, Isle of Luing**	Community café and heritage centre offering employment and training for local young people	Staff: Running the café, administration, organisation of events and training Volunteers: Board membership, helping to organise events Service users: Visiting café and heritage centre, taking part in social/leisure activities	Providing service for residents of one island (approximately 200 people, 14.2 km^2^) and visitors from surrounding areas.	Alternative service available on the mainland (1 h car journey with ferry crossing, limited public buses).
**Cantray Park, Croy**	Education, training and employment for adults with learning disabilities (café, market garden, wood workshop, animal aviary)	Staff: Running the departments, administration Volunteers: Board membership Service users: Receiving work experience and training, taking part in social/leisure activities	Service provision inclusive of adults with learning disabilities (aged 18 + years) across Inverness-shire rural areas. Population demographics only available for wider Highlands area, not Inverness-shire.	Main alternatives for education and skills development for adults with learning disabilities in main city of Glasgow (3-h drive in a car or 4-h train journey).
**Helmsdale and District Development Trust, Helmsdale**	Community development and support (development of infrastructure, environment, communication and economy)	Staff: Administration and operations support Volunteers: Board membership Service users: Wider community recipients of community development improvements	Serving the community of Helmsdale and the wider district with a population of approximately 700 people (Highland Council, 2011 figures).	No alternative. Limited public transport to the area.

The social enterprises studied catered for a wide range of age groups in their activities. The characteristics of communities in which the social enterprises were located corresponded with a ‘typical’ picture of challenges faced by rural communities, as described in the literature review. As shown in Table 1, social enterprises included in the study were filling gaps in service provision where alternatives were lacking or completely unavailable. Unlike in many urban areas, the rural communities under study were not surrounded by easily accessible community groups or leisure activities, therefore could not exercise choice over their participation in such activities.

### 3.3 Study sample

From the seven social enterprises, we interviewed 68 social enterprise stakeholders including social enterprise board and staff members, volunteers and service users who lived within each rural community.

Considering the research questions posted in this paper, data presented here focus on 35 participants who reported social isolation and loneliness before taking part in social enterprise activity. The sample characteristics are shown in Table 2.

**Table 2:** Participant characteristics.

Participant characteristics					
Gender	Male	Female			
All participants (n = 68)	25	43			
Study participants (n = 35)	16	19			
**Age**	**20**–**30**	**30**–**40**	**40**–**50**	**50**–**60**	**60+**
All participants (n = 68)	10	11	8	3	36
Study participants (n = 35)	7	2	0	1	25
	**Board**	**Staff**	**Volun-teers**	**Service Users**	**Total**
**All participants (n** = **68)**					
Seaboard Hall	3	5	4	9	**21**
Cothrom	0	5	1	0	**6**
COPE Ltd	0	5	1	5	**11**
Transport for Tongue	4	2	3	4	**13**
Atlantic Islands Centre	2	3	0	1	**6**
Cantray Park	0	3	1	3	**7**
Helmsdale DDT	2	2	0	0	**4**
**Total**	**11**	**25**	**10**	**22**	**68**
**Study participants (n** = **35)**					**Total**
Seaboard Hall	3	2	4	8	**17**
Cothrom	0	1	0	0	**1**
COPE Ltd	0	0	1	2	**3**
Transport for Tongue	2	3	0	4	**9**
Atlantic Islands Centre	0	0	0	1	**1**
Cantray Park	0	0	0	3	**3**
Helmsdale DDT	1	0	0	0	**1**
**Total**	**6**	**6**	**5**	**18**	**35**

As shown in Table 2, the majority of participants reporting social isolation and loneliness were service users, and were over 60 years old. The high proportion of older people is reflective of the population demographics in each location (e.g. a high level of retirees). Similarly, the high proportion of female participants compared to males reflected the high number of females working and volunteering at each of the social enterprises. The highest proportion of participants included in the study were from the Seaboard Hall social enterprise which had the largest number of stakeholders compared with other organisations.

### 3.4 Research method

A three-year study took place between 2015 and 2018 involving in-depth interviews between April 2016 and September 2017 with staff, volunteers and users of the social enterprise services. In-depth interviews allowed flexibility to probe areas of health and wellbeing in a flexible, unstructured and descriptive manner. Furthermore, it allowed for a conversational style of interviewing that was particularly suited to informal rural social enterprise settings, and when engaging with service users with additional needs (Yeo et al., 2013).

Interviewing a range of social enterprise stakeholders allowed for a broader exploration of the health and wellbeing effects of social enterprise activity from different perspectives. A snowball and convenience sampling approach was used, which is particularly useful when approaching populations where it may be difficult to identify target participants (Bryman and Bell, 2007), and where participants may not be readily available (Etikan et al., 2016). Initial key contacts were sought at each social enterprise, who then helped to locate relevant stakeholders, in particular, service users as the main beneficiaries of social enterprise activity.

Ethical approval for the study was provided from Glasgow Caledonian University. Before commencing interviews, a participant information sheet outlining the nature of the study, and a consent form, was given to each participant. Participants were informed that their individual names would not be used in research outputs but the name of the social enterprise would be included, therefore, anonymity could not be guaranteed as participants could be identifiable based on the activities they described. Interviews took place at social enterprise stakeholder's homes and workplaces to meet with participant's needs, availability and mobility. Interviews lasted approximately 1 h, and explored the nature of the participant's involvement in the social enterprise, how being involved in the social enterprise may have affected the participant's life in general and how the social enterprise activities may have impacted on specific areas of their health and wellbeing.

All interviews were audio recorded and then stored onto a password encrypted folder and laptop. Interviews were transcribed and uploaded into the qualitative data analysis software tool, NVivo. Data sorting firstly used structural and descriptive coding techniques to identify and segment data relating to the objectives of the study, and to code the data by topic under specific headings (for example, demographics, social enterprise activity, physical health). Secondly, the data was organised using pattern coding where similar or duplicated code topics were merged into larger headings and subheadings (Saldaña, 2016). The analysis then focused specifically on identifying emergent and prominent themes around health and wellbeing, and the social enterprise related processes that led to health and wellbeing impacts. These processes included ‘mediating factors’ that showed the mediating relationship between social enterprise activity and health and wellbeing outcomes; and ‘intermediate’ and ‘long-term’ outcomes that described the short and longer term linear pathways of social enterprise activity on health and wellbeing (as shown in Fig. 6). Themes and processes were then discussed with the research team, where feedback was received and consensus was sought.

## 4 Findings

In this paper, we present findings that relate to the role of social enterprise activity in addressing social isolation and loneliness in rural communities. For that reason, the following findings section will only report on the 35 research participants that reported social isolation and feelings of loneliness before their involvement with a social enterprise.

### 4.1 Characteristics of social isolation and loneliness in rural locations

Participants reported that their feelings of social isolation and loneliness had resulted from the following factors:

• Living alone: *through bereavement, separation and old age*
• Poor social connectedness: *a lack of similar interest groups or common links*
• Poor physical connectedness: *lack of transport, distance from others*
• Having nothing to do: *boredom, having a lack of choice of activities and entertainment*
• Being an incomer: *for example, a retiree, not integrated into a new community*

Living alone and having poor transport links have been previously identified in literature as contributing to social isolation and loneliness (McCann et al., 2017; Public Health England, 2015). Further, studies have previously described how temporal phases can have an effect on situational feelings of loneliness, such as, losing a partner, or moving to a new area (Shiovitz-Ezra and Ayalon, 2010). Yet, our findings showed further complex and intertwining issues around physical and geographical isolation that served to exacerbate factors contributing to feelings of loneliness or social isolation in these rural contexts. For example, ‘having nothing to do’ was not only interlinked with factors related to poor transport links, but also to a lack of choice of services and a lack of similar interest groups.

Living alone was a prominent theme relating to feelings of loneliness reported by participants over the age of 60 years, typically through the bereavement of a partner or spouse:

*‘I never use the word ‘lonely’, but I've got to admit to it to myself, I do have lonely days … I can sit in my house every day without seeing anybody, without speaking to someone’ (Male service user 1, Seaboard Hall, 60+)*

Social isolation and feelings of loneliness from living alone were felt to be worsened by having a house in a remote and sparsely populated location, and living away from a main road or settlement:

*‘October and April you could be sitting in the house all day and you would probably see one car. It can be that quiet’ (Male volunteer 1, T4T, 60+)*

*‘it's a bit different living out entirely on your own … I say you don't really make friends up here. I mean I know all the people here, but they're not going to come all that way (off the road) to visit are they?’ (Female service user 1, T4T, 60+)*

This meant that without the use of a car, participants were often hindered from visiting friends or acquaintances in their area due to poor public transport links:

*‘Unless you know somebody who's got a car, you are reliant on public transport that doesn't exist. And there isn't any. There is no train. The nearest train station is 40 miles away. There's no taxi. Taxis won't come out here this far’ (Female volunteer 1, T4T, 60+)*

The withdrawal of regular public buses also meant that many participants could not access employment, healthcare and amenities, or access social events.

*‘If you don't drive you're really stuck … it really impacts kind of getting a job as well, it's really hard to get a job, you know, if you don't drive and the buses are really slow or don't come at all’ (Female staff member, Cothrom, 20-30yrs)*

Participants also reported feeling socially isolated and lonely from having ‘nothing to do’ or not having a reason to get out of the house:

*‘What I've not been able to do (since moving in with my daughter for care purposes) is going to the theatre, eating out in nice restaurants or cafes for afternoon tea and coffee. I always did that, all my life. I would be surrounded by that kind of past-time hobby (in the city), things that I'm not doing here’ (Female service user 1, Seaboard Hall, 60+)*

Feelings of social isolation and loneliness from having ‘nothing to do’ were also linked to a lack of social connectedness to others. Small population sizes within communities mean that some felt unable to find companionship through common interest or circumstance. For example, young adults (aged 20–30yrs) with learning disabilities who were interviewed did not know any other adults with disabilities living in their area to share experiences with, until they started attending a social enterprise.

*‘Since leaving school I don't have any friends of my own age. Basically where I live I hang around with the younger children, and being able to be in that environment (Cantray Park) where there is the older people is basically what I need’ (Male service user 1, Cantray Park, 20-30yrs)*

A prominent factor for social isolation and loneliness was being an ‘incomer’ to an area, for example, having moved from a city to a rural location for retirement, or moving to a small rural community for a specific job role. Participants reported feelings of social exclusion from rural communities due to their incomer status from residents that had lived there their entire lives:

*‘When you're an incomer into an area people aren't really accepting, I don't think, especially in a small community. Maybe it’s my perception but you think, well, who does she think she is, she's only in the door five minutes' (Female service user 2, Seaboard Hall, 60+)*

Although social isolation and loneliness from moving to a new area may have been a temporal phase, the geographical dispersion of settlements of housing, and a lack of centralised amenities made it difficult for incomers to find opportunities to meet new people and socialise.

### 4.2 Social enterprise activity addressing social isolation and loneliness

The social enterprises involved in the study delivered a range of activities for the community that were counteractive to each of the risk factors for social isolation and loneliness. Although the range of activities provided were varied, interviewees described social enterprises as mediators for:

• *providing an increased reason or motivation to get out of the house*
• *providing increased opportunities to meet new people and interact with others*

On a fundamental level, the social enterprises provided a place to go or a means of getting there, therefore providing access to purposeful and meaningful activity:

*‘To actually have somewhere that people can congregate or to come every day at the same time every day and just have that kind of normality and something to do, something to look forward to’ (Male staff member 1, Atlantic Islands Centre, 20-30yrs)*

The social enterprises were addressing gaps in provision (e.g. leisure, transport, education opportunities) by providing services that were accessible, inclusive, and covering wide geographic areas. This was then directly or indirectly providing opportunities to form social relationships or gain social support.

Social activities such as taking part in leisure events, or visiting a social enterprise café, were viewed as being a vital source of social interaction for community members. Opportunities for social interaction were especially important for those socially isolated due to **living alone** in a remote location:

*‘It's helped me a lot because as I say, if I didn't have the hall here I'd be just sitting in the house watching television … I'd have nowhere to go … how can I put it, it's something I don't know what I'd do without it’ (Male service user 1, Atlantic Islands Centre, 60+)*

Organisations that provided specific social spaces, such as the Seaboard Hall or Atlantic Islands Centre, were reported to encourage the formation of social bonds and friendships. This was through regular interaction with other service users, volunteers and staff members:

*‘If I hadn't gone down to the social enterprise (Seaboard hall), I would be at home all day on my own … so, today, I've gone out, I've gone out with my friends for lunch, at the Seaboard hall and I've met, probably about ten or fifteen other people there today. Whereas I wouldn't have done, I would be at home all day on my own’ (Male service user 2, Seaboard Hall, 60+)*

**Fig. 2:**
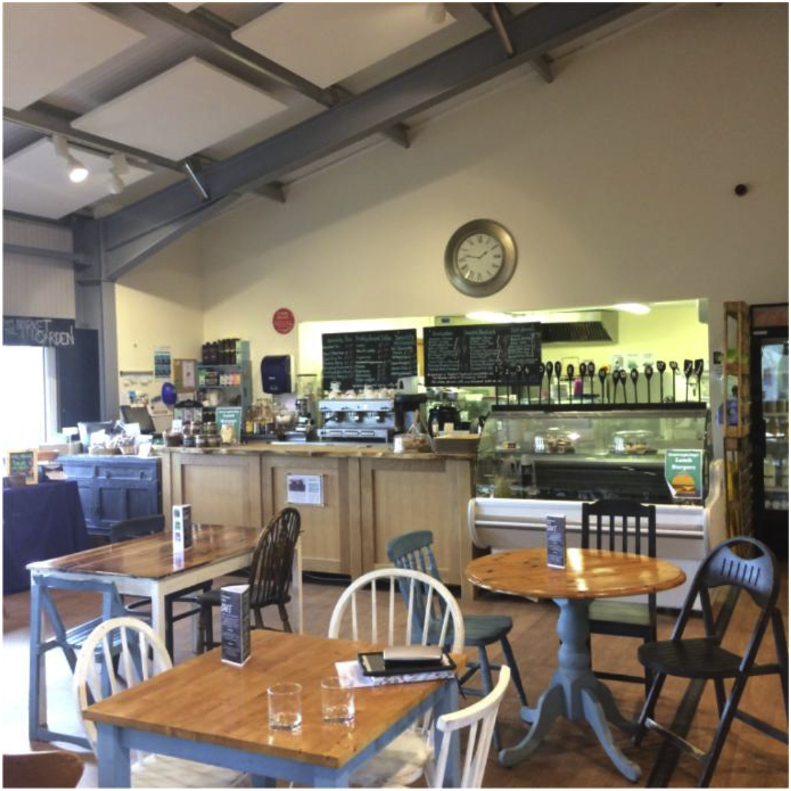
Café at cantray park.

The social enterprises Cothrom, COPE Ltd and Cantray Park (see Fig. 2) provided practical life experience skills and meaningful activities for disadvantaged adults, or those with physical and learning disabilities who reported having **poor social connectedness**:

*‘It gives people a place to go really, people with disabilities in a lot of areas don't have the opportunity to work, so to speak, and they're so proud to come here and work. Downstairs one of the participants loves speaking to the customers and getting involved and it gives them a sense of being’ (Female staff member 1, COPE Ltd, 30-40yrs)*

Although the social enterprises were not specifically aiming to provide social activities, they did offer multiple opportunities for social interaction with others, leading to the formation of friendships and bonds. This was particularly important for adults with learning disabilities who had previously lacked opportunities to engage with others:

*‘I meet new friends. I meet new people. I've got new friends and I met new friends that everyday I've never ever seen before. And then I meet new staff as well. And then I meet all different people that I've never seen before’ (Female service user 1, Cantray Park, 20-30years)*

**Fig. 3:**
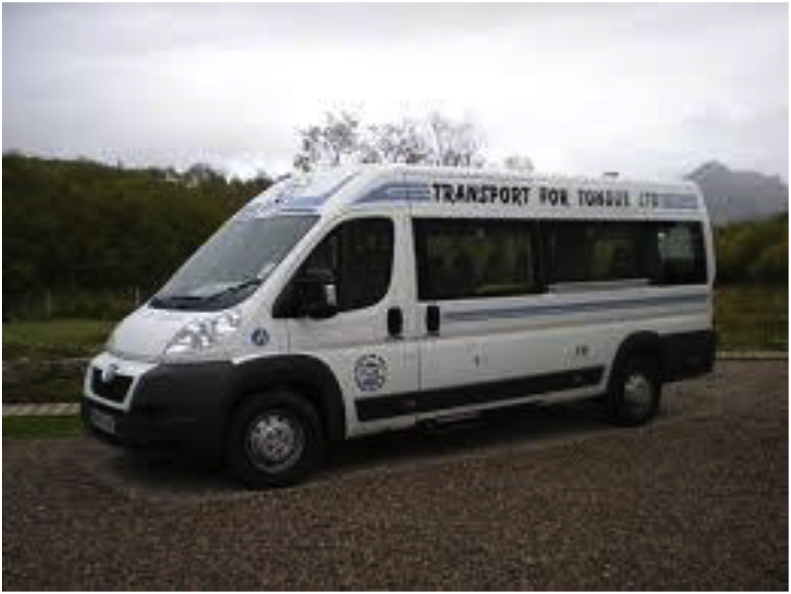
Transport for tongue community bus, tongue.

The social enterprise Transport for Tongue provided a mini-bus and car share service (see Fig. 3), transporting community members from their houses around the local area, and to large towns and cities. This meant that those who had felt **poor physical connectedness** could be connected to shops, social events and groups, healthcare, and education and employment opportunities outside of their immediate geographical region.

Older community members with limited mobility viewed this service as a *‘life-saver’* (Female service user 2, T4T, 60+):

*‘(Being picked up) and coming to this club, you know, this is the only day usually get out’ (Male service user 1, T4T, 60+)*

*‘It needs to carry on for older people because what you find up here is there's more older people than there is young people, you know. And a lot of people live in places way off the road, and they see nobody’ (Male volunteer driver 2, T4T, 50–60)*

T4T was not only addressing geographical isolation and connecting people physically, but also providing opportunities for social connections both on and off the bus, with journey times to towns and cities being up to 2 h long:

*‘It (the journey) gives them something to look forward to, you know. A major event, they're away to Inverness [a regional capital city], and they've got people at the back of the bus, and they're all talking away to each other, they get their lunch together’ (Male volunteer driver 2, T4T, 60+)*

Social enterprises were providing opportunities for **integration of incomers** who had just moved into rural areas. T4T provided new members of the community the opportunity integrate through volunteering, or simply using the bus service:

*‘(Having just moved to the area) if we weren't involved with T4T we would be a lot more isolated than we are, we would be socially excluded (from the community)’ (Male board member 1, T4T, 60+)*

*‘I've only been here 3 years, you see, so I already feel part of the community (because of T4T) … I also think you lose a bit of confidence, as you get older because you don't like to push yourself. Whereas here, you're very quickly into the community because of that (T4T) you see’ (Female volunteer 2, T4T, 60+)*

Participants reported that as a result of taking part in social enterprise activity their temporary feelings of isolation and loneliness had dissipated. The Seaboard Hall offered new people in the community a place to meet and connect with other community members in a space created at a central location in the village:

*‘you meet so many people because all the local people use the hall … I think we would have felt quite isolated because I didn't know anybody. My husband works away so he wasn't around … I think it would have taken us a lot longer to settle in to the area if we hadn't had the facilities here’ (Female service user 3, Seaboard Hall, 30-40years)*

The provision of a central, accessible meeting place in the community found to be a particularly vital source of community connection for older people who had moved to the area and were living on their own.

**Fig. 4:**
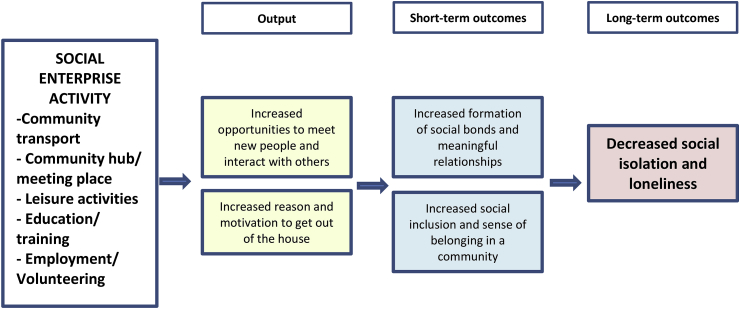
Diagram of pathways to decreased social isolation and loneliness from social enterprise activity in rural locations.

Based on the analysed data, Fig. 4 shows the key links between social enterprise activity and social isolation and loneliness. As shown, outputs are defined as the mediating factors that link social enterprise activity to short and long-term outcomes. For example, as a result of having increased opportunities to meet new people at the social enterprise, participants reported feeling an increased sense of belonging to a community, which in the long-term led to a decreased sense of social isolation and loneliness.

Findings showed that as a result of feeling a decreased sense of social isolation and loneliness through participation in social enterprise activities, participants reported further health and wellbeing impacts.

### 4.3 The health and wellbeing impacts of decreased rural social isolation and loneliness

Findings showed that participants had a decreased sense of unhappiness as a result of having regular interaction at the social enterprise and through the formation of new friendships:

*‘To start off, meeting people, even though it's just for an hour, it brightens me up because I get fed up watching television, because of my age, well, all my pals are all gone (Male service user 1, Atlantic Islands Centre, 60+)*

*‘It gives me an interest so that I don't sink into oblivion. As I say particularly because I was a widow … it fills a void for me’ (Female volunteer 1, HDDT, 60+)*

One service user reported that in having an opportunity attend the social enterprise Cantray Park, and an increased sense of social connectedness with young people with learning disabilities, this had a profound effect in decreasing his depression:

*‘After I left school I was basically just in my house doing nothing and basically I was getting depressed, very depressed, that I basically got to a stage where I basically wanted to kill myself. But obviously then I started here and basically I'm a lot happier’ (Male service user 1, Cantray Park, 20-30yrs)*

An increased sense of happiness and contentment came from regular socialisation; often framed in terms of having a sense of purpose and meaning to their lives from having something to do, and a means of accessing local SE services:

*‘I do find that getting out, even if it's only once/twice a week, it does help. You feel as though you've got a little bit more point to your life than sat indoors day in day out ‘(Female volunteer 1, Seaboard Hall, 60+)*

Participants also reported that their confidence levels had increased from having the ability to regularly converse and make social connections, where they had not been able to previously:

*‘I feel much more confident, I feel that I can talk to people, that I've met new people’ (Female staff member 2, Seaboard Hall, 30-40yrs)*

Service users in two of the social enterprises (Seaboard Hall and Atlantic Islands Centre) reported that as an in-direct result of visiting the organisations and having something to do, they had also decreased their negative health behaviours:

*‘I would say it's (the centre) kept me going. I hate doctors and hospitals. I had a bad problem with smoking and drinking and my daughter converted me onto this (the centre) and drinking just stopped altogether’ (Male service user 1, Atlantic Islands Centre, 60+)*

*‘I started to take an interest, after I stopped smoking … fill the time and the space, instead of going out for a cigarette, go out and do something positive than smoking … I did come here a lot more to get away from going out of the back and having a cigarette.’(Female service user 3, Seaboard Hall, 60+)*

The Seaboard Hall offered social leisure activities that were physical based, such as a bowls clubs. Users of the hall reported that because of taking part in such activities they had also increased their physical movement and decreased their sedentary behaviour, as they were no longer sitting in their house all day alone. This had led to an improved level of physical health, and in some older participants a decreased sense of frailty.

Taking these wider findings into consideration, Fig. 6 visualises the pathways from being involved in social enterprise activities to the health and wellbeing of participants. Social enterprise activity is presented as the ‘intervention’ that acts upon factors contributing to reduction of social isolation and loneliness among rural community members. Like in Fig. 4, mediating factors that link social enterprise activity to intermediate and long-term outcomes are described. The outcomes of social enterprise activity were found to lead to positive effects in the long-term on participant's self-reported physical and mental health, such as increased happiness and improved mobility.

**Fig. 5:**
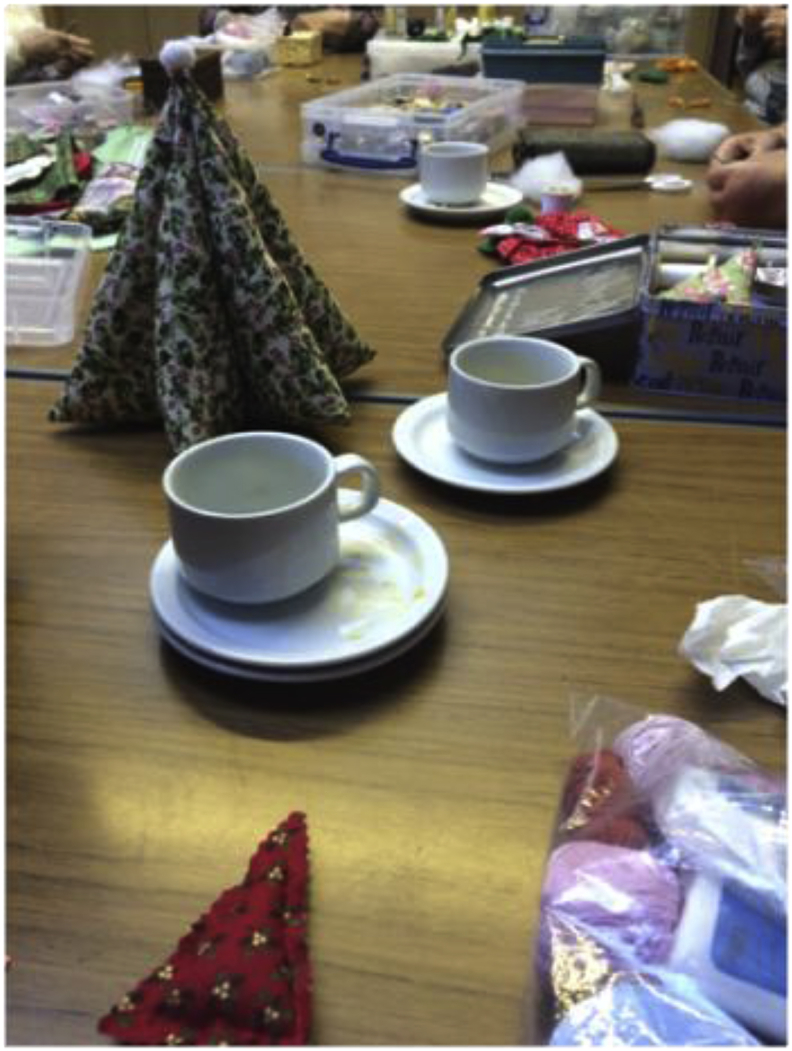
Arts and crafts morning at the Seaboard Hall.

For example, as a result of attending arts and crafts classes at a social enterprise (see Fig. 5), a female service user from the Seaboard Hall reported that she had increased opportunities to get out of the house and socialise with others, which had made her feel less lonely.

*‘It makes me feel good. It makes me feel better knowing I would just vegetated in the house alone just sitting there. The best part about spending time here, it's interacting with everybody … I'm one of them that like to talk, so I find it's good for me to come over and get a chat with someone … before the Seaboard Hall you were stuck, there was nothing.’(Female Service User 3, Seaboard Hall, 60+)*

**Fig. 6:**
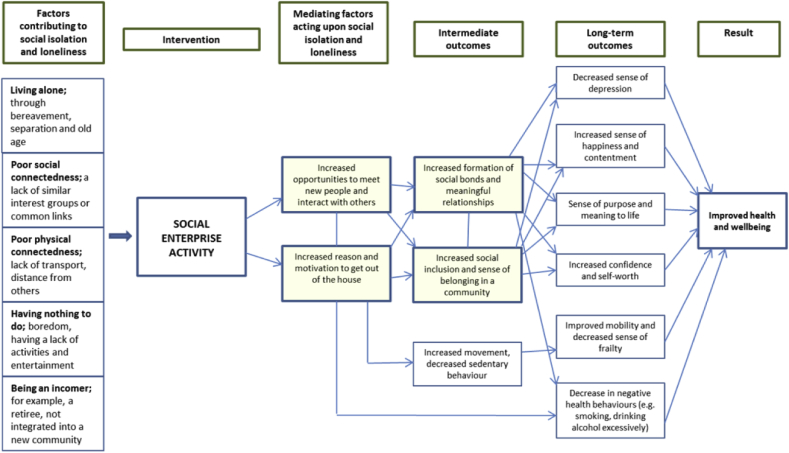
Pathways in which social enterprise activity acted upon health and wellbeing as a result of decreasing rural social isolation and loneliness.

In attending the arts and crafts class, she had not only gained confidence from opportunities to meet new people, but also in improving her sewing skills.

*‘Now I know how to sew even a button on, whereas as simple as that, I couldn't do that. So to me that's a big confidence thing, I used to say “I can't. I can't”. But now I can.’ (Female Service User 3, Seaboard Hall, 60+)*

As a result of increasing her social networks and confidence levels, the interviewee referred to an increased sense of happiness and contentment in her life.

Presented findings evidence the positive impacts of social enterprise activity in decreasing social isolation and loneliness in rural communities, and improving the health and wellbeing of social enterprise beneficiaries. However, findings also showed that in delivering and sustaining social enterprise services in rural areas, staff and volunteers faced negative health and wellbeing consequences.

### 4.4 Negative impacts on health and wellbeing of social enterprise staff and volunteers

The rural context often meant that there were smaller workforce pools to draw from. Furthermore, the nature of social enterprise activity meant that there was often a lack of funding to be able to pay staff, with a reliance on grants, donations and volunteerism. This was found to add pressure and burden on small numbers of existing community members to deliver services with limited economic and human capital:

*‘It's important to stress that we are all volunteers. Two of us are retired, the rest are in full time employment, so it is quite a big ask for all of us … everyone has their own lives' (Female board member 1, HDDT, 60+)*

As a result, staff members and volunteers reported feelings of ‘burn-out’ relating to exhaustion from the pressure of sustaining services, and working long hours without any time off:

*‘It was affecting my health, in that I was getting quite run down. Quite often, I would be going to work having sore throats and all manner of colds and things. And I knew it was because I was overworking’ (Female board member 1, Seaboard Hall, 60+)*

Having smaller population sizes also meant that rural communities were lacking the knowledge, skills entrepreneurship needed to run a social enterprise, therefore, having to utilise the limited skills available:

*‘There's not a full bank of folk out there where we can get whoever we like, so you kind of have to take who you've got here’ (Female volunteer 1, Atlantic Islands Centre, 50-60yrs)*

For this reason, members of the social enterprises reported an added feeling of risk in trying to run a formal organisation;

*‘When you're not trained to do this … you're making decisions and you're thinking, “Oh’’ your kind of basing it on your household income and think, “Well we can't afford to pay for that and we're not having that,” and you're doing that with this whole project of hundreds of thousands of pounds, which is scary’ (Female volunteer 1, Atlantic Islands Centre, 50-60yrs)*

To add further complexity, declining rural population levels, particularly on the islands, were leading to a decline in service users of social enterprises. This was reported to be problematic for the trading aspect of social enterprise activity, for example, when relying on income from customers visiting a community café or attending educational classes. Social enterprise staff and volunteers also reported that they lacked the confidence and skills to be able to write grant funding applications.

The rural populations that were studied all had a high level of over 60 year olds. Of these, most were retirees who did not want to continue working or volunteering, or had restricted physical ability. This was a particular problem for the community transport organisation, T4T, who relied on volunteer drivers to sustain their services:

*‘We're an ageing population, and basically you are relying on volunteers for the organisation. Either they are retired or too old … Most volunteer drivers are now too old to drive, their licenses will be pulled with no one to take over. Unless you are willing to shell out a lot of money for a special older person's license then you've got to be under 70’ (Female volunteer 2, T4T, 60+)*

Therefore, the continuity of their service delivery was questionable without the investment of younger volunteers, which was felt to be a ‘huge gap to fill’ (Female volunteer 2, T4T, 60+).

With smaller population size and closeness of rural communities, came an added feeling of emotional attachment to the social enterprise, and a personal responsibility and pressure to provide and sustain services. This was especially visible in communities where vital state services had been withdrawn. Staff and board members in particular reported that a feeling of increased visibility within their community as an ‘active member’, leading to an increased sense of responsibility when things went wrong:

*‘You think, “Oh gosh, this place might collapse and it will be my fault and everybody'll know it.” So I did feel the added pressure at that point to try and make things better, to try to keep this place going … we cannot just walk away’ (Female volunteer 1, Atlantic Islands Centre, 50-60yrs)*

Staff and volunteers at social enterprises also reported that working at a social enterprise had negative impacts on their personal lives, particularly those who were already retired:

*‘It does affect your family life, there's no doubt about it. Even going away for family weekends and that, sometimes you have to just miss out, because you've made a commitment, and it does impact on your social life, because you end up not having a social life’ (Female staff member 2, Seaboard hall, 60+)*

The negative impacts on social enterprise staff members and volunteers also meant that community members would be reluctant to volunteer, as they would often hear stories about the associated pressures and stress.

## 5 Discussion

The objectives of this paper were to investigate the role of social enterprise in addressing social isolation and loneliness in rural communities; and, explore the pathways in which social enterprise activity may act upon the health and wellbeing of social enterprise beneficiaries. This paper also further explored the capacity of rural community members to deliver and sustain social enterprise services. The evidence presented in this paper provides empirical grounding to the conceptualisations of social enterprises as generators of health through there varied remit of activities that impact on the social determinants of health, such as transport and employment opportunities (Nyssens, 2007, 2014; Ridley-Duff and Bull, 2015; Roy et al., 2014). Therefore, findings from this study, using the example of social isolation and loneliness, have moved this topic beyond the conceptualisation stage.

Although only a small number of social enterprises were studied, and we select data from users reporting social isolation and loneliness, the findings provide an important contribution to knowledge. We have shown *how* rural social enterprises are addressing social isolation and loneliness, even where this may not be their main purpose. Activities that are provided by rural social enterprises are directly or indirectly counteracting factors that are contributing to social isolation and loneliness, especially factors that are exacerbated by rural contexts, such as living in a sparsely populated area with poor transport links. Further, these organisations exist in areas where public service provision may have been withdrawn or non-existent, such as education facilities or public transport.

Although services and amenities in urban areas may be more plentiful, this study does not serve to detract from the fact that urban inhabitants can still experience feelings of loneliness and isolation. What it does outline, however, is that unlike in urban areas, the rural communities under study have also lacked choice in their ability to participate in activities or access services because of their geographical location and distance from towns and cities. Social enterprises have provided activities to counteract the lack of services and access to amenities affecting rural communities, and thus contribute to strengthening and sustaining these communities. Such activity has been structured around offering places for people to go, and giving them a means to get there. This has provided individuals with an increased reason or motivation to get out of the house, and increased opportunities to meet new people and interact with others, where they may not have previously had this option.

Fig. 4, Fig. 6 have shown that in providing opportunities to form social relationships and have access to support networks, rural social enterprise increase feelings of happiness and contentment in life among service users. Increased opportunities to interact and converse with others also leads to improved confidence levels. Having ‘something to do’ and a place to go led to individual's reporting an increased sense of purpose in live, and indirect physical health improvements, such as decreased alcohol use and improved mobility.

Although our study has presented evidence of the positive impacts of social enterprise activities on service users, our findings also indicate negative impacts of rural social enterprise on the health and wellbeing of service providers, such as staff and volunteers. This twofold impact of social enterprise activity on staff and volunteers is problematic. There are tensions and challenges between the willingness of participants to provide such services, and the burden and responsibility of sustaining an organisation. The study has shown that the continuity of rural social enterprises is under strain due to smaller workforce pools and a reliance on volunteerism, leading to burnout and stress, a lack of formalised knowledge and entrepreneurial skills within declining populations, and the pressure of serving the needs of the community. These findings contradict the policy rhetoric that encourages the ‘empowerment of communities to tackle social isolation and loneliness’ (Scottish Government, 2018a:13), without any consideration of the capacity of community members in rural settings to generate health.

## 6 Conclusion

Presented evidence shows that rural social enterprise can play an important role in addressing social isolation and loneliness experienced by rural communities. New knowledge is also generated through identifying pathways in which rural social enterprise activity may act upon the social determinants of health that effect the health and wellbeing of social enterprise beneficiaries. Rightly, social enterprises may be recognised in policy as being well placed to address social isolation and loneliness as they are often rooted within and led by communities, and have the ability to offer more flexible alternatives to statutory services. However, this study has also shown that relying on social enterprise as a solution to social isolation and loneliness, and wider health and wellbeing concerns is precarious due to complexities associated with rurality.

In light of this, policy measures must consider the sustainability of these solutions and the support that is required for their continuation. Most notably, funding and skills enhancement support is required to ease the stress on volunteers and staff members in the daily running of social enterprises. Approaches to tackling social isolation and loneliness cannot be a ‘one size fits all’, and policy must accommodate local level complexities and contextual differences, such as low workforce pools and declining populations. In particular, the capacity for rural communities to provide alternative services that may have been withdrawn by the state, such as public transport, should be considered.

Our findings open new research avenues for future studies on rural social enterprise and health. Further research is required to explore social isolation and loneliness in other international rural contexts to verify presented findings and test identified pathways in which rural social enterprise enhance health and wellbeing as a result of decreasing rural isolation and loneliness. Such information is required to inform future policy on social isolation and loneliness. Social isolation and loneliness is presented as one example of how social enterprise activity is impacting on the health and wellbeing of rural communities. Further academic research would benefit from an exploration of aspects of mental and physical health that social enterprise activity may address in rural contexts, such as depression or mobility.

## Declarations of interest

None.

## Sources of funding

This work was jointly supported by the Medical Research Council and the Economic and Social Research Council [grant number: MR/L003287/1].

## Footnotes

1 Social enterprises are third sector organisations that are engaged in trading activities with a social mission, whose trading surpluses are then reinvested into social activity, rather than distributed to owners and shareholders (Department of Trade and Industry, 2002).

2 http://www.commonhealth.uk/.
